# Implementation of Radiating Elements for Radiofrequency Front-Ends by Screen-Printing Techniques for Internet of Things Applications

**DOI:** 10.3390/s19163626

**Published:** 2019-08-20

**Authors:** Imanol Picallo, Hicham Klaina, Peio Lopez-Iturri, Aitor Sánchez, Leire Méndez-Giménez, Francisco Falcone

**Affiliations:** 1 Department of Electric, Electronic and Communication Engineering, Public University of Navarre, 31006 Pamplona, Spain; 2 Department of Signal theory and Communications, University of Vigo, 36310 Vigo, Spain; 3 Institute for Smart Cities, Public University of Navarre, 31006 Pamplona, Spain; 4 Lan Printech, Centro Tecnológico Miguel De Eguia, 31200 Estella, Spain

**Keywords:** conductive screen-printing, internet of things, flexible substrates, wireless sensor networks

## Abstract

The advent of the Internet of Things (IoT) has led to embedding wireless transceivers into a wide range of devices, in order to implement context-aware scenarios, in which a massive amount of transceivers is foreseen. In this framework, cost-effective electronic and Radio Frequency (RF) front-end integration is desirable, in order to enable straightforward inclusion of communication capabilities within objects and devices in general. In this work, flexible antenna prototypes, based on screen-printing techniques, with conductive inks on flexible low-cost plastic substrates is proposed. Different parameters such as substrate/ink characteristics are considered, as well as variations in fabrication process or substrate angular deflection in device performance. Simulation and measurement results are presented, as well as system validation results in a real test environment in wireless sensor network communications. The results show the feasibility of using screen-printing antenna elements on flexible low-cost substrates, which can be embedded in a wide array of IoT scenarios.

## 1. Introduction

The progressive adoption of Internet of Things (IoT) is expected to open new opportunities in a wide range of markets, spanning from home automation to smart health, industry 4.0 or connected vehicles, among others. This will lead to a sustained increase in the number of devices connected to the Internet, exceeding 30 billion in 2020, with global IoT expenditure of almost 800 billion dollars [1]. IoT systems are formed by a set of elements in order to provide interactivity, which are [2]:

▪ identification (naming and matching services with demand)
▪ sensing (data gathering for transmission towards data warehouse and/or single board computing devices)
▪ communication (connection of devices to enable data transmission, usually with low power requirements and flexible quality of service demands)
▪ computation (capability to perform signal processing tasks, which can be performed locally or make use of cloud computing or edge computing capabilities, depending on local device constraints)
▪ services (categorized as a function of the application, e.g., identity related, information aggregation, context awareness and ubiquity, among others)
▪ semantics (knowledge extraction and information analysis for adequate service provisioning and planning)

The implementation of the aforementioned IoT elements requires the use and integration of multiple systems and technologies that need to consider inherent limitations in terms of size, energy consumption, processing capabilities and cost. This is leading to constant research efforts in order to provide feasible large scale IoT scenarios, considering aspects such as interference coordination, efficient bandwidth allocation and interoperability by means of cognitive radio mechanisms [3], distributed confidence based on the application of technologies such as Blockchain [4], optimization of information searching techniques for specific IoT applications [5] or the implementation of purpose specific IoT platforms, such as Industry 4.0 [6] and others.

Among the IoT elements, communications play a key role in order to provide user/device interaction capabilities, as well as to transmit data for business intelligence and data analytics purposes. Wireless communications are widely employed in order to provide mobility/ubiquity, with multiple systems employed depending on coverage/capacity requirements [2]. In this way, transceivers embedded in IoT applications can make use of different communication protocols, combining wide coverage technologies such as public land mobile networks (2G to 4G systems, including machine to machine communications based on NB-IoT or M-Cat), local area networks (such as WiFi-802.11 protocols), body area networks (such as Bluetooth/Bluetooth low energy or near field communications) or wireless sensor networks (such as ZigBee, LoRa, eNOcean or IQRF, among others). In this sense, wireless transceiver and radio frequency (RF) front-end design face limitations of cost, size and adaptive capabilities. Antenna systems (including radiating elements, feed lines and matching networks) exhibit specific design challenges, as radiation characteristics are strongly affected by elements such as device material, shape and size. This is particularly relevant in the case where conformal or embedded antennas are required, such as biomedical applications, vehicular communications or smart city contexts.

One of the advances in this sense is the use of flexible substrates to implement different types of electronic systems and radiating elements. The evolution in fabrication processes and availability of different flexible low-cost materials (such as polymide–Kapton, polyetheretherktone–PEEK or polyethylene therephtalate–PET) has opened the path to employ flexible electronics in multiple domains, such as consumer electronics, energy storage systems, solar cells or biomedical systems, among others [7,8,9,10]. More specifically in the case of radiating elements, different approaches to implementing antennas on flexible substrates have been explored, such as the use of liquid metals for 2D [11] and 3D [12] antennas, the use of flexible microwave grade substrates with conventional machine milling techniques to implement conformal antennas [13,14,15], the use of textile substrates [16] or the use of conductive fibers [17]. Radiating elements can also be implemented by making use of the deposition of conductive/functional inks and pastes, using inkjet approaches, in which silver or silver chloride inks are employed owing to the balance between high conductivity, deposition feasibility and certain biocompatibility [18,19,20,21,22,23,24,25,26]. Radiating elements have been implemented using inkjet techniques for low cost Radio Frequency Identification RFID applications on paper substrates [19,20], flexible plastic substrates such as Kapton [21], radiating elements over ultra-thin substrates [22], complex radiating elements based on fractal patterns [23], millimeter wave flexible antennas [24] or volumetric antennas and lenses based on the inkjet fabrication process. The proposed studies, however, do not exploit the use of screen-printing techniques for the implementation of radiating elements within the microwave frequency range, nor are system level tests performed on the proposed prototypes.

In this work, radiating elements will be designed, implemented and tested, based on screen-printing of conductive inks on low cost flexible substrates. Fabrication conditions will be described, indicating the impact of different elements, such as substrate/ink election, drying temperature or substrate angular deflection. Individual device performance will be analyzed, as well as wireless channel characteristics in terms of propagation losses and system level behavior when operating in a real wireless sensor network based on ZigBee protocol.

The paper is organized as follows: Section 2 describes the screen-printing and radiating element implementation process; Section 3 describes the characterization of the implemented prototypes; Section 4 presents wireless channel characterization, as well as system level results on a real wireless sensor network; Section 5 provides a discussion on the results and Section 6 presents the conclusions.

## 2. Description of the Screen-Printing Process and the Implementation Procedure of Antenna Prototypes

**Figure 1:**
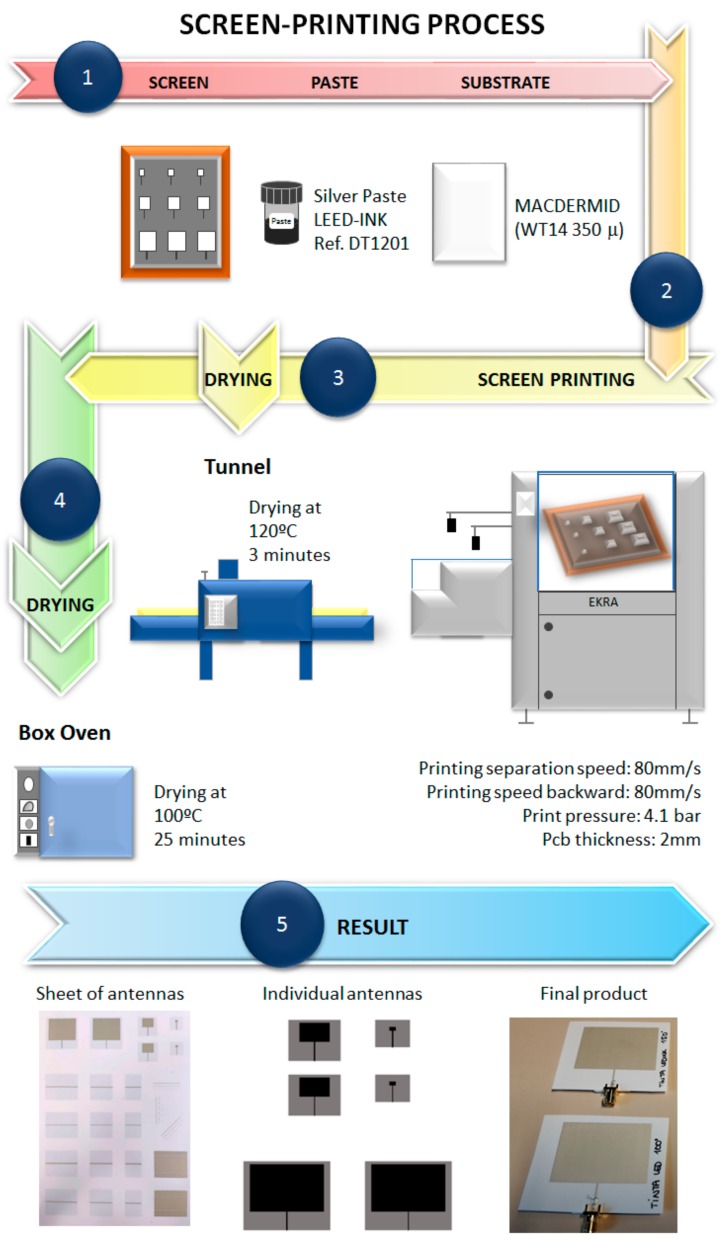
Schematic description of the fabrication of antenna prototypes based on the screen-printing process, divided into five steps: (**1**) material parameter election, (**2**) printing process, (**3**) tunnel drying, (**4**) oven drying, (**5**) sheet prototype and subsequent processing.

The proposed approach for the implementation of antenna prototypes on flexible substrates is based on the use of a screen-printing process, which is schematically depicted in Figure 1. The process is based on the deposition of a uniform layer of conductive/functional ink paste on a flexible substrate, usually based on paper or plastic. The employed inks/pastes in conventional screen-printing processes are usually based on silver or silver chloride, due to the adequate balance they offer in terms of cost, conductivity and flexibility in the deposition process [26]. In this work, the following commercially-available conductive pastes were employed: Dupont, Ref. 5874, silver/silver chloride paste; Leed-ink, Ref. DT1201, Silver Paste; Mateprincs, Ref. SCAG-002, silver paste. The paste is deposited into the substrate by applying pressure with a mechanical palette, which displaces the conductive paste onto the host substrate with a pattern that is given by a stencil that is located between the deposition head and the host substrate. The precision of the final implemented layouts printed in the host substrate depend on stencil characteristics, given mainly by wire density and emulsion thickness. Based on lab trials, the employed stencils were designed with 130.30 wires per inch and an emulsion of 25 µm thickness. Once the stencil has been designed and fixed, the parameters, which are shown in Table 1, are fixed in order to perform the printing process (i.e., printing speed, printer head palette pressure, spacing, etc.). Parameter selection is mainly given by the operational characteristics of the printer, which is a EKRA E2 series 900703 serigraphy printer. After careful alignment of the stencil and the substrate, the printing process takes place.

**Figure 2:**
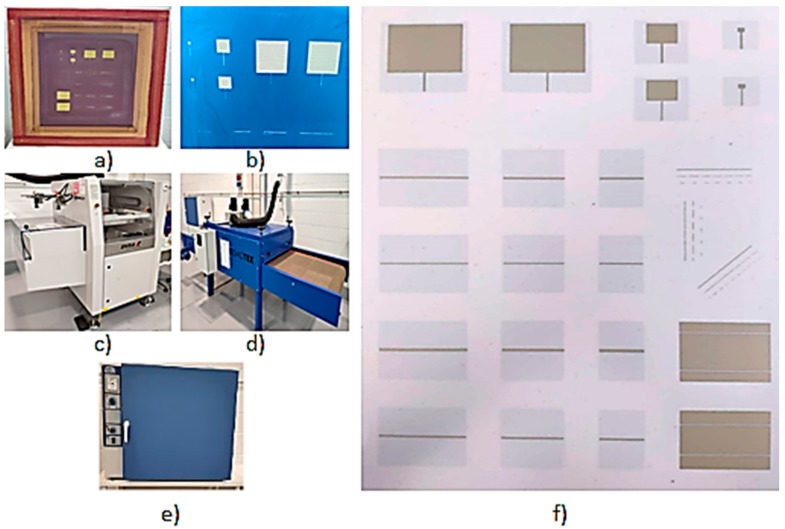
(**a**) Printing stencil, (**b**) detail of the implemented mask, (**c**) screen printer, (**d**) tunnel, (**e**) box oven, (**f**) printed sheet (top layer) as an output from the aforementioned screen-printing process. Multiple layouts can be placed within the sheet, for calibration and test purposes. The tested prototypes can be seen on the top right of the image.

Once the printing process is completed, the result is a sheet with multiple layouts with conductive/functional ink deposition. An example of the printed sheet and the employed elements within this work is depicted in Figure 2. If the circuit has more than one layer, then the process must be repeated for each corresponding layer. In this work, the radiating elements are implemented in microstrip technology, i.e., two layers are printed (top layer with the access transmission line and radiating patch and bottom layer, with the corresponding ground plane). The sheet has to be dried in order to consolidate the conductive ink layers, hence avoiding blurring of the printed geometries. Printed prototype drying is a two-fold process: first, drying takes place in a tunnel (model Cosmotex), in which prototypes are placed in a conveyor belt (with a displacement time of 3 min); second, the printed sheets are taken to a box oven (forced air, model Selecta 2000201), where they will be placed for a longer period of time (in this work, 25 min). The parameters in the drying process depend mainly on the characteristics of the conductive inks employed, as this process has a direct impact on the final dimensions of the prototypes (owing to the thermal expansion coefficients of both the substrates and especially the conductive inks) and indirectly on conductivity values (owing to homogeneity values of the silver particle distribution in the ink emulsion). The values employed are depicted in Table 1, based on practical experimentation and vendor specifications.

Once the sheets have undergone the two-fold drying process, the prototypes are ready to be manipulated. In this case, individual antenna and transmission line elements must be separated and cut away. As the individual antenna elements are prepared, connectors must be included in the transmission line terminal of the prototype. The connector inclusion process will be discussed in more detail in Section 3.

## 3. Flexible Antenna Prototype Results

**Figure 3:**
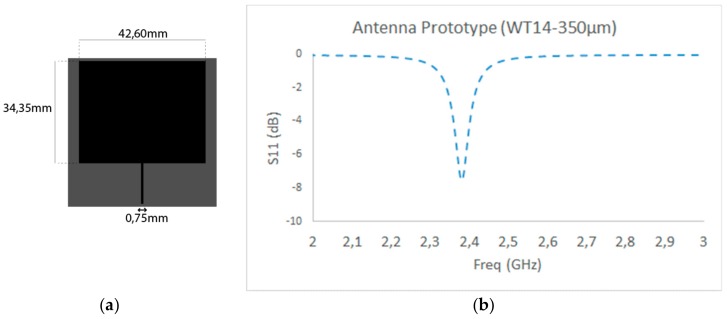
(**a**) Dimensions of the microstrip antenna prototype; (**b**) the S11 parameter full wave simulation result (WT14 350 µm substrate).

**Figure 4:**
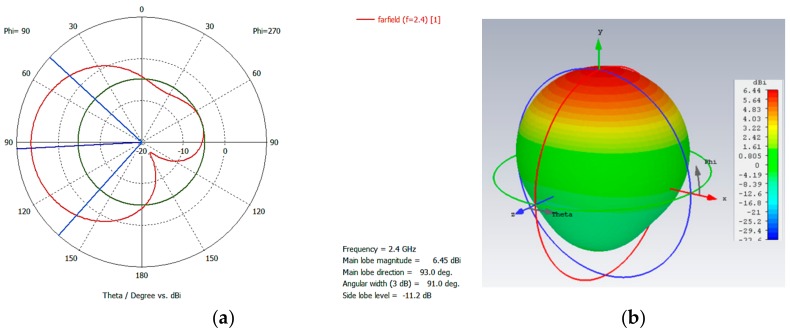
(**a**) 2D radiation diagram and (**b**) 3D radiation diagram for the WT14 350 µm antenna prototype.

Once the characteristics of the screen-printing process, as well as the specifications of the different components, have been described, radiating prototypes based on functional ink on flexible substrates are designed and implemented. The design procedure is based on planar microstrip antenna parameters, in which dimensions of the microstrip patch are obtained in order to have a fundamental radiation frequency at 2.4 GHz, and the transmission line parameters are fixed in order to have an input impedance of 50 Ω [27]. The employed substrates are plastic or paper based, with a substrate thickness in the range of 175–350 µm (except Valox FR1 with a thickness of 500 µm) and dielectric constant ($\epsilon_{r}$) values in the range of 2.6–3.3. Based on the analytical expressions obtained from consideration of the dominant cavity resonant mode, the dimensions of the patch, as well as of the transmission line (in this case to comply with the matching condition *Z_in_* = 50Ω), are obtained. An example of the dimensions used for the case of the WT14 substrate, with 350 µm thickness and $\epsilon_{r}$ = 2.9, is depicted in Figure 3. These results were confirmed by full wave electromagnetic simulation, obtained with CST MW Studio^TM^, where the matching parameters (e.g., the S11 parameter) and the radiation patterns were computed and are shown in Figure 3 and Figure 4. The resulting resonant frequency is approximately 2.39 GHz, a deviation corresponding to the fact that the initial analytical estimation does not fully consider the effective dielectric constant of the microstrip patch antenna, which is modified by the fact that the dominant mode is a quasi-TEM mode, in which part of the electromagnetic fields propagate in the surrounding air volume, besides the host dielectric substrate [27].

**Figure 5:**
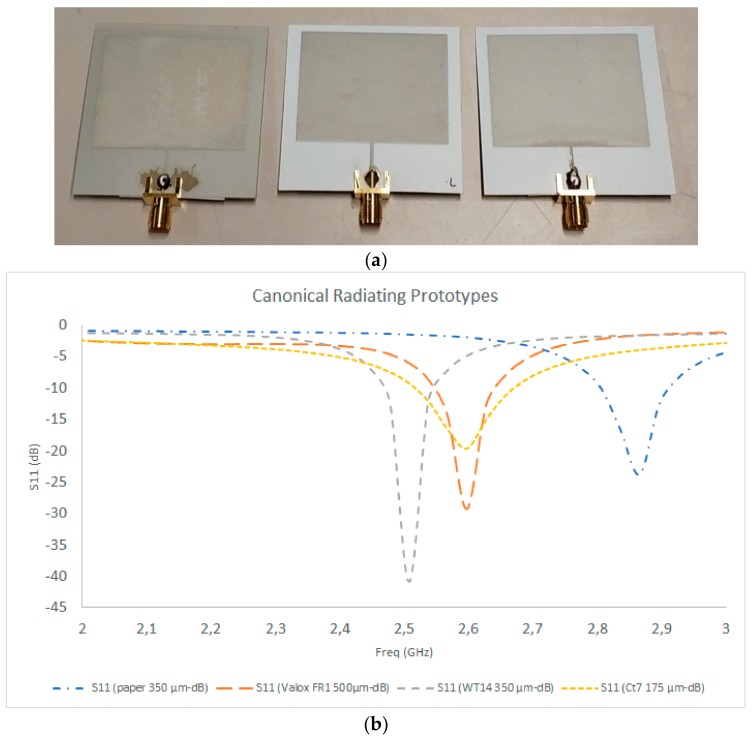
(**a**) Fabricated prototypes for radiating elements; (**b**): S11 parameter measurement results for different substrates (plastic/paper).

**Figure 6:**
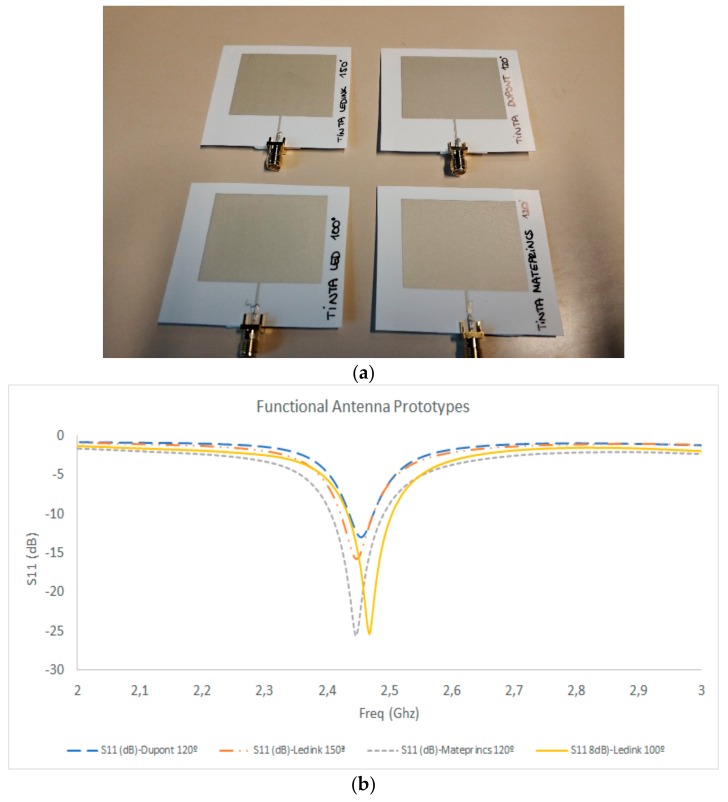
(**a**): Fabricated prototypes for different ink types and temperature conditions. (**b**): S11 parameter measurement results.

Radiating elements on different substrates were fabricated based on the screen-printing process described in the previous section, and are depicted in Figure 5, along with the measurement results for the S11 parameter. It is worth noting that connectors must be included in the transmission line endpoint in order to provide access to the vector network analyzer measurements, which require a 3.5 mm D SMA coaxial connector. Commonplace connectors are usually microstrip launchers, which require soldering of the top connector pin to the microstrip access transmission and the bottom portion of the connector to the ground plane of the antenna. The use of flexible substrates hinders this process, as soldering degrades the substrate and also provides a mechanically unstable connection. To solve this issue, the connectors were bonded both to the transmission line and to the ground plane with the aid of conductive adhesive paint, as well as with mechanical fitting elements between the connectors and the substrate. As can be seen from the measurement results from the S11 parameters, good matching was observed for all of the prototypes, with resonant frequencies in the range of 2.5 GHz to 2.85 GHz. The frequency shifts are mainly given by differences in the values of the dielectric constants between substrate batches (usually in the range of 1%–10% difference in dielectric permittivity among different batches), as well as dimensional changes within the heating process, depending on the host material employed. The results show that the best performing substrate is WT14 with a substrate height of 350 µm, so further prototypes were implemented using this substrate. Figure 6 shows the measurement results for antenna prototypes in which different inks were employed. The results show that the resonance frequency is in the range of 2.44 GHz to 2.46 GHz, with matching levels from −12 dB to −25 dB. The best results were obtained for the case of Mateprincs and Leed Ink, which were the prototypes that were adequate for real operational use.

**Figure 7:**
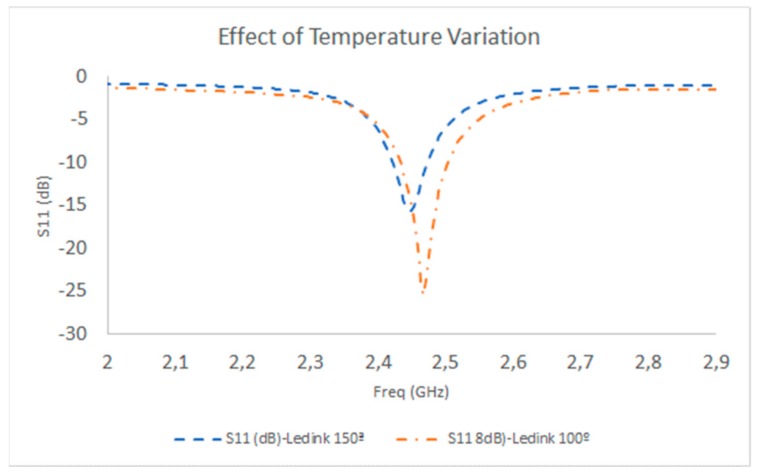
S11 parameter measurement results (WT14 substrate prototype), as a function of temperature variation in the prototype curing process.

**Figure 8:**
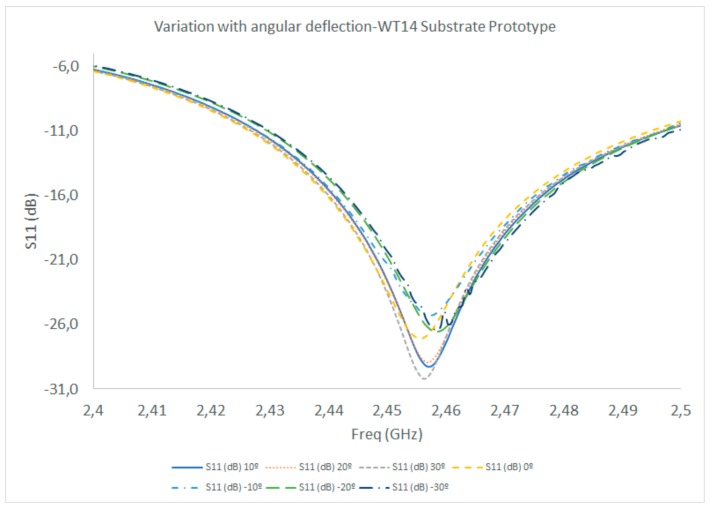
S11 parameter measurement results (WT14 substrate prototype), as a function of angular deflection of the substrate, within the −30° to +30° range, with respect to the horizontal plane containing the antenna.

In order to analyze the impact that the fabrication process and the use of flexible substrates have on device performance, measurement tests were performed considering variations both in the drying temperature and the angular deflection of the substrate. Measured S11 parameters obtained from the prototype connection to the Vector Network Analyzer (VNA) are depicted in Figure 7 for temperature variation in the drying process, and in Figure 8 for the case of angular deflection. In the case of temperature variation, the temperature of the box oven was modified in the range of 100° to 150°. The impact of temperature modification is mainly related to the dimensions of the antenna prototype, given by the thermal expansion coefficients of the employed ink and of the host substrate. Moreover, drying temperature also plays a relevant role in consolidating the conductive paste to the substrate; if the temperature is too low (usually below 80° C), the paste is unstable and gets blurred. The results in Figure 7 show that the resonance frequency is closer to the design frequency in the case of a lower temperature (100 °C) with a higher frequency shift, with better matching in the case of a higher temperature (150 °C).

In the case of angular deflection, the measurement results were obtained when the antenna prototype was bent from its original configuration, where 0° was the initial angular value with respect to the horizontal plane in which the antenna prototype was contained. Angular deflection was produced by exerting force with a pair of dielectric tweezers located at the edges of the prototype, without touching the conductor patch. The results are depicted in Figure 8 and the maximum matching levels are shown in Table 2. As can be seen, the matching levels are always better than −25 dB, with a slight frequency shift (1 MHz over the central operating frequency), in a deflection range that spans from −30° to +30°. These results indicate that the antenna prototypes have conformal capabilities, enabling their integration in multiple configurations, devices and embedding conditions.

## 4. Wireless System Validation

Once the antenna prototypes based on screen-printing have been characterized, their performance is tested in a realistic wireless sensor network configuration, providing system level validation. In order to do so, wireless channel behavior is characterized with the aid of a deterministic channel estimation based on the 3D ray launching technique and radio channel measurement results. Once this characterization has been performed, the prototypes are embedded in a ZigBee wireless sensor network and packet level analysis is performed under real operation.

**Figure 9:**
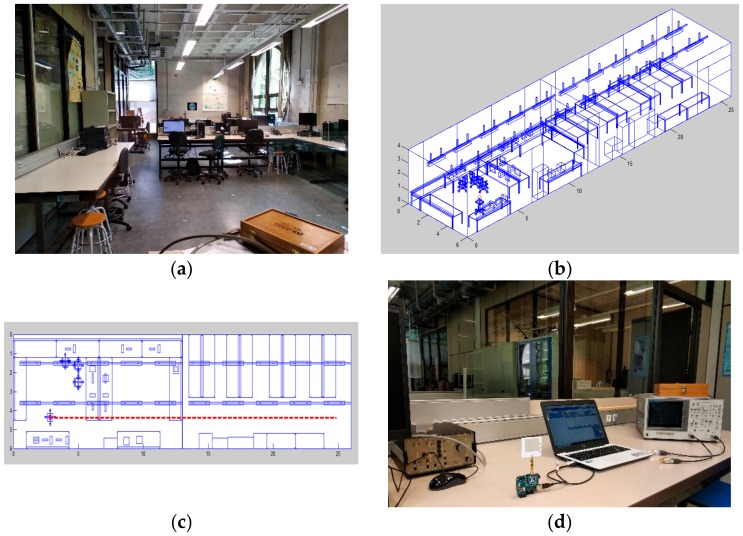
Lab scenario employed for system level validation: (**a**) Real lab environment, (**b**) volumetric scenario implemented in the 3D Ray Launching simulation tool, (**c**) detail of the radial validation layout, and (**d**) detail of the measurements with the ZigBee mote network.

The initial step of wireless channel characterization is to estimate the received power levels, which determine the coverage/capacity relations by comparing the aforementioned estimations with the corresponding receiver sensitivity levels, given by parameters such as required bit rate, employed modulation and coding scheme and hardware specifications, such as noise factor values. The characterization was obtained by deterministic wireless channel modelling and was validated with single carrier continuous wave measurements. The scenario employed is the Luis Mercader laboratory at Universidad Pública de Navarra (UPNA), depicted in Figure 9. The scenario has multiple rooms separated by glass/plywood walls, as well as doors and multiple objects and furniture inside. Wireless channel simulation is based on the 3D ray launching technique, based on the combination of geometric optics with a uniform theory of diffraction. The simulator was implemented in-house, and was extensively tested and described [28,29]. The basis of operation relies on discretizing the electromagnetic wave front in equivalent rays launched from the transmitting source, following a solid angle distribution, and following the propagation direction of the wave vector. The number of launched rays depends on the angular resolution of both the horizontal and vertical planes, whereas the numerical resolution is also given by the size of the cuboids in which the volumetric scenario is meshed, as well as by the maximum number of allowed reflections until a launched ray is extinguished. Each one of the rays follows Fresnel equations when interacting with the elements within the scenario (reflection, refraction and diffraction). These interactions in turn strongly depend on the material location, shape and frequency-dependent behavior.

**Figure 10:**
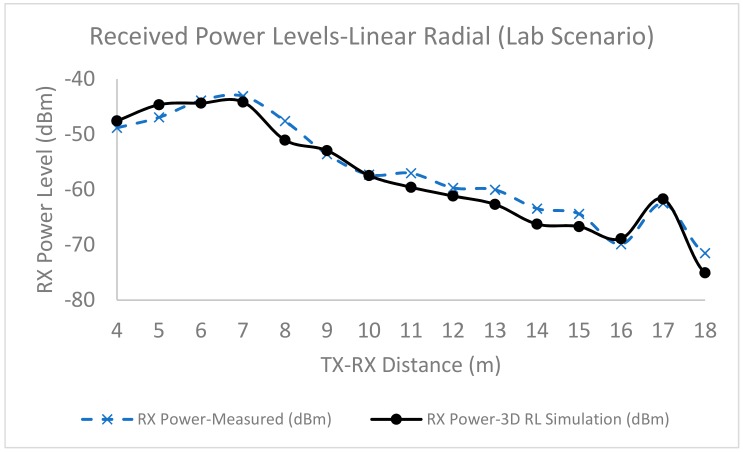
Comparison between simulation and measurement results of the received power levels for a linear Transmitter-Receiver radial within an indoor lab environment.

**Figure 11:**
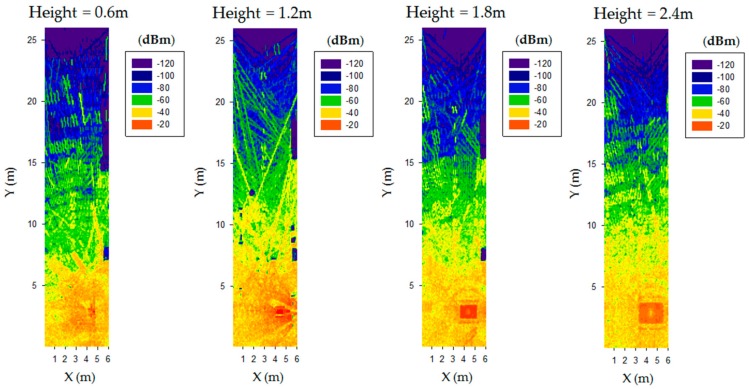
Estimation of the received power levels, obtained with the in-house 3D ray launching simulation. Results were obtained for the complete volume of the scenario and have been particularized for the case of 2D height planes of 0.6 m, 1.2 m, 1.8 m and 2.4 m.

From the previous comment, it was necessary to employ volumetric simulation models of the scenario that consider the location of objects, their corresponding shapes and the material employed for each one of those elements. Therefore, a volumetric lab scenario (Figure 9b,c) was generated in Matlab in order to perform 3D RL simulations, considering objects (location, shape, size and frequency dispersive material parameters). The employed simulation parameters were chosen following extensive convergence analysis and calibration of the 3D RL code, as detailed in [30] and are detailed in Table 3. Figure 10 shows the simulation results compared with the measurement results for a single carrier continuous wave source (implemented with a voltage controlled oscillator operating at 2.4 GHz), following the linear radial depicted in Figure 9c. As can be seen, there is good agreement between both the simulation and measurement results, with values above the usual sensitivity levels in the case of wireless sensor network transceivers (in the range of −90 dBm). Extensive wireless channel simulations were obtained for the complete scenario volume. An example of bi-dimensional coverage distributions as a function of different heigth cut-planes (which can be given by the application of the potential nodes, which can be embedded in elements such as furniture, closets, and lighting) is depicted in Figure 11. Height variations imply changes in received power level distributions, indicating the impact of elements within the scenario, even if relative sizes are not very large, owing mainly to the multipath components generated to scatterers present within the scenario.

Once the wireless channel behavior had been analyzed and modelled, the system validation tests were performed. A ZigBee network based on Arduino One boards connected to Digi Xbee shields and motes was programmed and implemented. An image of one of the motes is seen in Figure 9d, which forms a point-to-point link (worst-case scenario, without activating meshing capabilities). A sequence of 1000 packets was programmed, and the received packets and acknowledgement frames in the transmitter node were studied. The transmitter was located in the initial transmitter position of the Voltage Controlled Oscillator (VCO) measurement, whereas the receiver node was displaced along different locations, spanning distances from 2 m to 20 m between the transmitter and receiver. For all locations and for all of the transmitted sequences, 100% of the packets were correctly transmitted and received, in line with the fact that the received power levels were always above the required sensitivity threshold. Similar tests were conducted, where the antennas for conventional monopoles (3 dBi gain at 2.4 GHz) were changed, showing equivalent results in terms of system performance.

## 5. Discussion

The antenna prototypes showed adequate performance in terms of resonance frequency and impedance matching in general for all considered substrates and conductor pastes. Optimal operation was seen when using the WT14 substrate with a thickness of 350 µm and Mateprincs or Leed Ink. In relation to substrate selection, radiating elements in the case of planar antenna perform better if the substrate thickness is greater, due to the larger equivalent resonating cavity [26]. However, increasing substrate height excites higher order transverse magnetic (TM) modes, which can propagate along the substrate by total internal reflection mechanisms given by the air-dielectric interfaces. These conditions imply power loss due to coupling from the fundamental q-TEM mode to those substrate modes. It is worth noting, however, that in the case under study, the substrate thickness values indicate that substrate mode coupling will take place within the millimeter wave range, not being particularly relevant for conventional applications in IoT that use wireless communication systems in general in the below 6 GHz range.

The fabrication process implies the use of high temperatures in the drying process, which produce dimensional differences owing to thermal expansion coefficients, mainly in the substrate material. Careful characterization, based on experimental knowledge of the fabrication process is compulsory in order to adequately fix the screen-printing parameter setting, which is strongly dependent on the material selection. Operational conditions, such as angular substrate deflection, were also tested, showing a good response for deflection angles in the range of −30° to +30°, indicating that the prototypes enable conformability as well as embedding capabilities. These properties are desirable for our aim of providing communication capabilities to a wide array of devices and objects, within IoT frameworks. In this sense, RF connector interfacing was implemented by using adhesive conductor paste and mechanical substrate insets with conventional 3.5 mm SMA microstrip launcher connectors. The proposed connector setup showed a good performance in terms of insertion losses over the period in which experimental tests were performed. The connector setup of the flexible substrate antenna prototypes is however an open research issue and future work will be devoted to exploring new alternatives, such as ad-hoc pressure connectors.

Wireless channel characterization, as well as system level results, showed that the proposed antenna prototypes perform adequately in real wireless sensor network scenarios. The tests were performed considering point-to-point links, a worst-case scenario for ZigBee networks, that enable meshing capabilities. Future work can be devoted to the implementation of more complex radiating systems, such as antenna arrays or multiband antennas that include elements such as subwavelength resonators or fractal geometries. It is worth noting that higher frequency bands can be considered as a function of the dimensional tolerances achievable in the screen-printing process, given mainly by the stencils employed, configuration of the ink paste deposition process and losses of the substrate material. Extension within the microwave band, such as 5 GHz Wi-Fi bands, is feasible in principle. Multiband operation is also in principle possible, within the frequency band of operation. The co-existence of multiple bands is limited by restrictions in frequency masks (i.e., potential interference of adjacent channels or interference), similar to other printed circuit approaches, such as mechanical milling or the chemical etching fabrication process.

The use of planar flexible substrates enables the implementation of low profile radiating elements, with conformal capabilities up to a certain angular range. As future work, it is necessary to consider the effect of including encapsulating elements, such as top plastic layers or varnishings, which add additional protection from environmental agents. Limitations can be given by the modifications in the input antenna impedance, which can degrade matching and hence, radiating system efficiency.

In order to provide more insight into the application of the screen-printing technique, the different approaches previously described are outlined and compared in Table 4. The main benefit in the use of screen printing techniques is the possibility to produce highly reproducible batches with good performance within the RF and microwave frequency bands of operation, in flexible substrates of different types.

## 6. Conclusions

In this work, antenna prototypes implemented on flexible substrates with conductive inks were presented. The prototypes were fabricated by means of the screen-printing process, in which different types of substrates as well as conductive inks were considered. The screen-printing process was described, as well as the required parameters for adequate prototype production, based on experimental knowledge of the fabrication process. Microstrip antenna prototypes were designed, simulated and measured, showing good response in terms of frequency of operation and impedance matching. Multiple prototypes were implemented in order to consider different substrate materials, as well as the use of different conductive inks. Fabrication effects, such as temperature variation in the drying process, were also shown. Angular deflection inherent to the use of flexible substrates was analyzed, indicating a robust response within a +/−30° range. Wireless channel characterization based on deterministic 3D RL estimations was compared with continuous wave frequency measurements, showing good agreement and signal levels above the sensitivity thresholds. System level measurement results were obtained for a ZigBee network implemented with Arduino One single board processors connected with Digi Xbee motes operating in the 2.4 GHz frequency band. The trials performed within a real test scenario showed correct reception for 100% of the transmitted packets, for all the locations within the scenario. The proposed antenna prototypes provide a low cost solution for embedded antenna systems, and find direct application in a wide range of IoT scenarios.

Evolution of the proposed technology will deal with potential frequency extension into higher frequency ranges, to cover systems such as Wi-Fi or below 6 GHz operation of 5G systems. Moreover, multiband antennas can be considered, in order to integrate the multi-system operation into individual radiating elements. Future works will also deal with improving the connector setup, providing a simpler and more stable connection, as well as studying the feasibility of integrating the radiating elements with shielding structures or radomes, in order to provide embeddable elements.

## Figures and Tables

**Table 1:** Screen-printing process parameters.

Process	Parameter	Value
Ink Printing	Printing separation speed:	80 mm/s
	Printing speed backward:	80 mm/s
	Print from:	130 mm
	Print to:	−90 mm
	Print pressure:	4.1 bar
	Pcb thickness:	2 mm
	Snap off:	0 mm
	Separation way:	0.1 mm
	Separation speed:	0 mm/s
	Print pressure:	4.1 bar
	Pcb thickness:	2 mm
Ink Drying		
LeedInk	Drying Tunnel	120º-3 min
	Oven	100º-25 min
Mateprincs	Drying Tunnel	130º-3 min
	Oven	100º-25 min
Dupont	Drying Tunnel	120º-3 min
	Oven	120º-25 min

**Table 2:** Matching performance as a function of angular deflection.

Δº	Resonance Freq (GHz)	|S11| (dB)
−30º	2,458	−26,5
−20º	2,458	−26,6
−10º	2,457	−25,3
0º	2,455	−27,1
10º	2,457	−29,3
20º	2,457	−28,9
30º	2,456	−30,2

**Table 3:** Parameters utilized for the 3D ray launching simulations.

Parameter	VCO + Spectrum Analyzer	ZigBee Motes
Transmitted Power level	8.38 dBm	7.99 dBm
Frequency of Operation	2.4 GHz	2.4 GHz
Antenna Gain (Transmitter)	5 dBi	5 dBi
Antenna Type (Transmitter)	Monopole	Monopole
Launched rays angular resolution	1 degree	1 degree
Maximum number of rebounds	6	6
Cuboids size	10 cm × 10 cm × 10 cm	10 cm × 10 cm × 10 cm

**Table 4:** Comparison of radiating elements.

Type-Ref	Frequency of Operation/Matching Level	Application
Liquid Metal (EGaIn)	3.45 GHz/−10 dB	Initial Test, not application specific
Liquid Metal [12]	885 MHz/−30 dB	Mobile Phone Integration
AMC [13]	2.45 Ghz/−25 dB	Wearable Devices
Flexible Kapton Array [14]	2–4GHz/−10dB	UWB Breast Imaging System
Textile Antenna [16]	2.4Ghz/−15 dB	Integration into garmets, wearable operation
E-fiber Textile [17]	915 MHz/−35dB	Harsh environments, vehicular applications
Inkjet Printing on Flexible substrates [19]	1 GHz/−20dB	RFID and WSN applications
Screen Printing on Flexible Substrates [This work]	2.4GHz/−30 dB	Enabling communication capabilities within IoT
